# Planning and Problem-Solving Impairments in Fibromyalgia: The Predictive Role of Updating, Inhibition, and Mental Flexibility

**DOI:** 10.3390/jcm14155263

**Published:** 2025-07-25

**Authors:** Marisa Fernández-Sánchez, Pilar Martín-Plasencia, Roberto Fernandes-Magalhaes, Paloma Barjola, Ana Belén del Pino, David Martínez-Íñigo, Irene Peláez, Francisco Mercado

**Affiliations:** 1 Department of Psychology, Faculty of Health Sciences, Rey Juan Carlos University, 28922 Alcorcon, Spain; roberto.fernandes@urjc.es (R.F.-M.); paloma.barjola@urjc.es (P.B.); belen.delpino@urjc.es (A.B.d.P.); david.martinez@urjc.es (D.M.-Í.); irene.pelaez@urjc.es (I.P.); francisco.mercado@urjc.es (F.M.); 2 Research Group in Cognitive Neuroscience, Pain and Rehabilitation (NECODOR), Rey Juan Carlos University, 28922 Alcorcon, Spain; 3 Department of Biological and Health Psychology, Faculty of Psychology, Universidad Autónoma de Madrid, 28049 Madrid, Spain; pilar.martin@uam.es

**Keywords:** chronic pain, dyscognition, executive functions, fibromyalgia, planning, problem-solving, predictive role

## Abstract

**Background/Objectives**: Fibromyalgia syndrome (FMS) is a chronic pain condition in which executive function (EF) alterations have been reported, though strikingly, relationships between simple executive functions (EFs) (updating, inhibition, and mental flexibility) and high-order ones, such as planning and problem-solving, have not been addressed yet in this population. This research aimed to firstly explore how low-level EFs play a role in planning and problem-solving performances. **Methods**: Thirty FMS patients and thirty healthy participants completed a series of neuropsychological tests evaluating low- and high-order EFs. Clinical and emotional symptoms were assessed with self-report questionnaires, while pain and fatigue levels were measured with numerical scales. Importantly, specific drug restrictions were accounted for. **Results**: Patients scored lower in most neurocognitive tests, with statistical significance noted only for visuospatial working memory (WM) and two planning and problem-solving tests. Pain, fatigue, and sleep disturbances showed important effects on most of the cognitive outcomes. Multiple regression analyses reflected that planning and problem-solving were successfully and partially predicted by updating, inhibition, and mental flexibility (though differences emerged between tasks). **Conclusions**: Our study confirms the presence of cognitive impairments in FMS, especially in high-order EFs, supporting patients’ complaints. Clinical symptoms play a role in FMS dyscognition but do not explain it completely. For the first time, as far as the authors know, simple EF influences on planning and problem-solving tests have been described for FMS patients. These results might help in unraveling the dysexecutive profile in FMS to design more adjusted treatment options.

## 1. Introduction

Being able to focus attention, mentally manipulate information, and solve problems are abilities commonly required to perform daily activities, especially when speaking about a successful adult life. Nevertheless, some clinical conditions (e.g., chronic pain) can negatively impact these cognitive processes, thereby reducing people’s capacity to function normally in their everyday activities. Pain has been described as one of those factors affecting cognition [1,2], though a better understanding of their mutual influence is still needed, particularly when considering the presence of chronic pain.

Fibromyalgia syndrome (FMS) is a chronic widespread pain condition with a global prevalence of 2.7% [3] and a prevalence of 2.45% in Spain, being more frequently found in women [4]. Beyond pain, patients show unexplained intense fatigue, non-restorative sleep, and cognitive complaints, often referred to by patients as *fibrofog,* whose impact on patients’ quality of life and functionality has been highlighted in several studies [5,6,7,8,9]. Over the last twenty years, there has been a vast amount of research aimed at finding objective support for *fibrofog* in neuropsychological assessments, and the most recent literature reports robust evidence for working memory (WM) and executive alteration [10,11].

Several neuropsychological investigations have highlighted the presence of impairments in higher-order executive functions (EFs), such as planning, problem-solving [12], and decision-making [13,14]. In particular, it has been reported that FMS patients made more mistakes and took longer than control participants when completing both versions of the Zoo Map Test (ZMT) [15,16,17] or the Key Search Test (KST) [12], tasks included in the Behavioral Assessment of the Dysexecutive Syndrome (BADS) [18] to measure planning or the ability to organize, sequence, and execute actions. Other instruments employed in the assessment of high-order EFs in which FMS samples underperformed healthy samples are the Tower of London (ToL) [12,19] and the Revised Strategy Application Test (R-SAT), where scores in the latter were interpreted as a poorer capacity for strategic planning and self-regulation ability [15,17]. FMS patients have also been reported to have a reward hypersensitivity in classic decision-making tasks such as the Iowa Gambling Task (IGT), where they made more disadvantageous elections [13,14]. Nevertheless, heterogeneous results in these cognitive processes have also been described [20]. In an effort to systematically review the literature, a recent manuscript [21] concluded that defects in EF in FMS seem to be consistent but also appeared to be associated with the simultaneous presence of chronic pain and other core symptoms. Pain intensity, worrying about pain, fatigue, depression, and anxiety have been shown to be related to worse performance in planning and problem-solving processes [15,16] as well as in decision-making tests [12,13,22]. Nevertheless, not all literature supports the relationship between these factors and cognition in FMS [15,22,23,24], suggesting that not everything about their mutual influences is known yet.

Interestingly, empirical studies and theoretical proposals have pointed out the idea that WM and other simple executive components may be critical when considering high-order EFs, acting as related and participating processes [25]. In accordance with this point of view, Miyake and colleagues [26] focused on updating, inhibition, and shifting, and authors explained that those principal components could be combined to support the initiation of more complex EFs [25,27]. In a similar vein, the hierarchical model of EFs posed by Adele Diamond [28] posits three analogous core EFs (inhibition, WM, and mental flexibility) that would be necessary to build the more complex EF processes such as planning and problem-solving. The simple executive processes mentioned by Miyake’s and Diamond’s models have been explored in some manner in FMS. Updating impairment has been demonstrated by lower outcomes when patients performed Digits Backwards [24,29], Spatial Span in the inverse order [30], n-back paradigms [12,31], or in the Paced Auditory Serial Addition Test (PASAT) [32]. On the other hand, a recent meta-analysis pointed out the existence of solid evidence on problems regarding inhibitory control in this chronic pain population [10], reflected in a variety of instruments measuring interference effects [33,34,35,36,37], go–no-go tasks [38], and other similar measures [33,34,35]. With respect to mental flexibility and shifting operations, some support exists about its impairment in FMS [19,24,39,40], though size effects seem to be small following the literature [10]. Controversial findings with respect to the neurocognitive performance in updating [32,35,39,41,42], inhibition [7,9,43,44], and mental flexibility assessments [9,33,35,39,45] suggest that more research is needed to better understand them in FMS patients. Furthermore, the interdependencies between these three components could be enabling the performance of higher-order cognitive functions; however, to the best of the authors’ knowledge, the relationship between both of them (simple executive components and higher-order functions) has not yet been comprehensively explored in FMS, nor the possibility that the former might predict the latter in some manner and, if so, how it happens. Nevertheless, this approach has been previously employed in the research of high-order EFs with college students [26,46], people suffering from mild cognitive impairment [47] and also in relation to academic performance [48] and science problem-solving [49] with children.

Therefore, the present study aimed to analyze whether the simple EF components proposed by Miyake’s model (updating, inhibition, and mental flexibility) can predict performance in planning and problem-solving tasks (higher-order functions). To achieve this goal, performance on different simple EF tasks (WM, inhibition, mental flexibility, verbal fluency, and dual task) and higher-order executive instruments (planning and problem solving) were contrasted between a group of healthy women and a comparable group diagnosed with FMS, additionally exploring the possible effect of relevant clinical variables (i.e., pain, fatigue, sleep disturbances, anxiety, depression, and worrying about pain).

## 2. Materials and Methods

### 2.1. Participants

A group of sixty women were enrolled in this study, 30 with a diagnosis of FMS and 30 healthy control participants (HC). FMS sample was recruited from local associations of patients while various recruitment strategies were used for healthy participants. An advertisement was published on a volunteers’ website where people offer and request voluntary activities (www.hacesfalta.org, accessed on 22 November 2010), in which a brief explanation of the purpose of the study and contact instructions were given. Informative e-mails asking for participants in the study were also sent to the social environment of the Faculty of Health Sciences at Rey Juan Carlos University. Finally, a local cultural association was contacted, as many of its members were women who met the participating criteria.

All participants met the general inclusion criteria, with ages ranging from 25 and 65 years old, being right-handed, having at least an elementary educational level and normal or corrected vision and hearing. Women were not allowed to participate if they suffered from a psychiatric illness or had a current diagnosis of major depression and/or anxiety disorder given by a medical specialist, and those presenting neurological impairments were also excluded. Special interest was paid to the pharmacological treatment participants were undergoing at the time of the study. None of them were taking anxiolytics, antipsychotics, neuroleptics, opioids, or any other drugs that could significantly alter cognition, with the exception of stable doses of non-steroidal anti-inflammatory drugs, non-opioid analgesics, and/or specific types of anti-depressants known to have low effects on cognition according to the literature [50,51,52,53,54,55,56] and to technical data sheets as defined by the Spanish Agency for Medications and Healthcare Products (selective serotonin reuptake inhibitors and dual anti-depressants or dopamine agonists). This procedure was previously used in other investigations [54]. Engaging in or having a history of consumption or drug abuse was another exclusion criteria, though limited use of tobacco (≤20 cigarettes a day) or alcohol (<3 drinks a day) was permitted. The final exclusion criterion was undergoing proceedings to assess incapacity to work at the time of the study.

Specific inclusion criteria for the clinical group included having a diagnosis of FMS given by a qualified medical specialist following American College of Rheumatology criteria [57,58] and not presenting a concomitant diagnosis of Chronic Fatigue Syndrome or Rheumatoid Arthritis. Healthy participants were admitted if they did not have an illness that caused chronic pain. The sociodemographic and clinical characteristics of both groups are displayed in Table 1. No significant differences between groups were found in age, educational level, Estimated Intelligence Quotient (IQ) (measured with Wechsler Adult Intelligence Scale III (WAIS-III) Subtests of Vocabulary and Block Design as the most frequently used method [59]), marital status, or tobacco and alcohol use. As expected, FMS participants consumed a significantly higher amount of non-steroidal anti-inflammatory drugs and non-opioid analgesics, and other drugs for common illnesses. Finally, FMS patients had a mean of 6.97 years since they received the diagnosis of their pain condition.

### 2.2. Procedure

The current study was approved by the Rey Juan Carlos University Research Ethics Board (registration number: 201000100011550), and it followed all the requirements from this committee. All participants were instructed about the procedure and provided written informed consent afterwards. Upon arrival at the Laboratory of Cognitive Neuroscience (Faculty of Health Sciences, Rey Juan Carlos University), a brief interview was separately held with each of participants to check that they met the inclusion and exclusion criteria. If none of the exclusion criteria were met, different appointments were scheduled to carry out clinical and neuropsychological assessments, which took place in an adequate room in the Faculty of Health Sciences at Rey Juan Carlos University. Clinical assessment lasted about 60 min and neuropsychological evaluation took about two hours. The tests included in both protocols were applied in the same order to each participant. Possible effects of fatigue were controlled by introducing short resting periods between the examination sessions. No economic compensation was given to any participant, but they were asked whether they wanted to be informed about the overall conclusions once the research had finished and data had been analyzed.

#### 2.2.1. Neuropsychological Assessment

A brief description of selected instruments to evaluate cognitive functioning is given below, organized by the cognitive processes they are intended to measure.

**WM maintenance, manipulation, and updating**: (1) Digit Span Forward (DSFW) and Backwards (DSBW). In this subtest from WAIS-III [60], participants had to listen to and repeat various series of digits forwards and backwards, as a measure of auditory–verbal WM. The total scores for each version and for the whole task were calculated. (2) Spatial Span Forward (SSFW) and Backwards (SSBW). As a part of the Wechsler Memory Scale-III (WMS-III) [61], this was used as a measure of visuospatial WM. The instruction was to tap different series of cubes in the same order as the clinician, forward or backwards. The total scores for the whole test and each version were summed. (3) Letter and Number Sequencing (LNS): From the WAIS-III, the instruction was to repeat a series of mixed numbers and letters in a specific order, providing a total score. (4) Paced Auditory Serial Addition Test (PASAT) [62]: Here, the subject was instructed to listen to numbers appearing every 3 s (PASAT 3″) or 2 s (PASAT 2″) and to sum each one with the number previously heard. The total correct responses were considered.

**Inhibition/Interference control:** Word and Color Stroop Test [63]. This is a well-known instrument where participants need to complete three tasks (reading words, naming colors, and saying the color of the ink in which names are written out loud, which inhibits reading them as the automatic response), enabling the calculation of the Stroop Interference Control (SIC) score.

**Mental flexibility/Shifting:** (1) Comprehensive Trail-Making Test (CTMT) [64]. Here, scores from Trails 4 (CTMT 4) and 5 (CTMT 5) were used. In Trail 4, the subject had to draw a line connecting numbers from 1 to 21 no matter how they were presented, whether in Arabic or as a written word. In Trail 5, participants had to link, in an alternating sequence, numbers and letters in increasing and alphabetic order, respectively (1-A-2-B-3-C…). The time employed in each task was recorded. (2) Wisconsin Card-Sorting Test (WCST) [65]. In this test, usually employed to assess mental flexibility and categorization ability, participants had to pair two decks of 64 cards with one of four possible given models that differed in color, shape, or number of elements as possible classification criteria, after receiving feedback from the clinician. We noted the total perseverative errors and failures to maintain the set as outcomes.

**Access to memory storage:** (1) Letter Fluency Test (FAS) [66]. This test was performed to measure access to lexical information; the instruction was to say as many words as possible starting with a letter (F, A, and S) in one minute. (2) Semantic Fluency (Animals) [66]. Participants were asked to name as many animals as possible in 90 s, as a variation of the traditional task.

**Dual Task (DT)**: We used an adaptation of the design by Tirapu-Ustárroz and Luna-Lario [67], and their correction steps. Firstly, participants had to copy the Rey–Osterrieth Complex Figure [68], then they were asked to name as many animals [66] as possible, and finally they had to perform both tasks simultaneously. Each part lasted 90 s.

**Planning and problem-solving:** (1) Key Search Test (KST) [18]. The instruction given was to think of a successful strategy to find lost keys inside a closed space presented as a square in a sheet of paper, as an evaluation of planning and behavioral monitoring as complex EF processes. The time employed and raw scores were recorded from this test included in the BADS. (2) Zoo Map Test (ZMT) [18]. As a part of the BADS, this test was used to assess planning and behavior regulation, comprising a less-structured version (ZMT1) where participants had to plan a visit to different sites in a zoo without breaking some given norms, and a more structured one (ZMT2) in which they just need to follow a list of instructions to guide their visit. The scores for each version and the total test were used for this study. (3) Tower of Hanoi (ToH) [69]. This task has been considered useful for evaluating problem-solving and planning abilities, as well as strategy selection and the monitoring of behavior. A platform with three posts is placed in front of the participant, with a tower of 4 (ToH4D) or 5 disks (ToH5D) in the left one. The objective was to replicate the tower in the right one, using the three posts if needed but moving only one disk at a time and making the fewest movements. The total number of movements for each version was registered.

#### 2.2.2. Clinical Assessment

**Pain and fatigue numerical scales** (PNS and FNS, respectively) from 0 to 10 were employed to evaluate pain and fatigue levels, asking participants to, respectively, rate their current level of pain and fatigue, with 0 representing an absence of pain and 10 the most intense sensation they could imagine.

**The Beck Depression Inventory** (BDI) [70] was used to assess depressive symptoms. It is a widely used self-report questionnaire that includes a total of 21 items with four response options ranging from 0 (absence of symptom) to 3 (most severe symptom). Participants were asked to choose the response that best reflects their mood, feelings, and thoughts over the past week. Total score (0–63) is calculated summing responses from the 21 items with higher results indicating more severity of depressive symptoms. The BDI has an internal consistency of 0.86 and a test–retest fiabilty of 0.70.

**The State-Trait Anxiety Inventory** in its Spanish adaptation (STAI-T) [71] was used, particularly the Trait form, which is composed of 20 items and quantifies the disposition to feel anxiety in a more general way. Total scores can vary from 0 to 60 points after participants evaluate their feelings using a four-rate scale from ‘Almost never’ to ‘Almost always’. Its internal consistency ranges from 0.84 and 0.87 for the Trait form.

**The Pittburgh Sleep Quality Index** (PSQI) [72] was employed to evaluate sleep disturbances. It is a 19-item self-report instrument designed to assess sleep quality and its alterations. In our study, we only used the sleep disturbances subscale following scoring indications from the test manual. Its internal consistency varies from 0.67 for students to 0.81 for a clinical sample.

Finally, the Pain Catastrophizing Scale (PCS) [73] was applied to measure pain related worrying. It is a 13-item scale, with five possible responses from 0 “None at all” to “All the time” that expresses different thoughts and feelings people can have when feeling pain. Total score ranges from 0 to 52 and is calculated by summing answers from all the items, with higher values reflecting greater levels of catastrophizing. It has a good internal consistency with a Cronbach’s alpha of 0.79).

### 2.3. Statistical Analysis

First, we proceed to normalize the data by transforming them to Z scores. Independent-sample *t*-tests (scaled variables) and Chi-Square tests (categorical variables) were conducted to compare sociodemographic and clinical variables among FMS and HC participants. Multivariate analyses of variance (MANOVAs) were computed on neuropsychological data to explore group discrepancies in the updating of information for WM (DSFW/DSBW, SSFW/SSBW, LNS, and PASAT), interference control (SIC), mental flexibility/shifting (CTMT 4, CTMT 5, WCST perseverative mistakes, and failures to maintain set), lexical access (FAS and Animals), DT paradigm, and planning and problem-solving (KST, ZMT1, ZMT2, ToH4D, and ToH5D). Additionally, analyses of covariance (ANCOVAs) were performed, entering pain, fatigue, sleep disturbances, depression, and trait anxiety as covariates, as their potential influence in cognition have been previously reported in FMS patients (see the Section 1). Effect sizes were exposed by adjusted eta square values (η^2^_p_) for those contrasts reaching statistical significance.

Critically, with the aim of studying the predictive value of simple EFs for planning and problem-solving, we performed statistical analyses in two steps. Initially, we executed a factorial analysis to confirm that our measures were actually grouped on the three simple executive processes proposed by Miyake’s model of EF (updating, inhibition, and mental flexibility) and, subsequently, we created indexes by summing the Z-scores of the neuropsychological instruments saturating in each factor. Then, we conducted multiple regression analyses separately for each group of participants, where the three indexes of simple EFs were introduced in the model as predictors and complex EFs (planning and problem-solving tests) were considered as the dependent variables.

All the statistical analysis described in this section was performed in SPSS Statistics 28 (IBM Inc., Armonk, NY, USA). Statistical significance was established at *p* < 0.05. Note that, though analyses were performed based on Z scores, some tables displaying neuropsychological performance show raw scores (means and standard deviations) for a better comprehension of the results.

## 3. Results

### 3.1. Group Differences in Clinical Measures

Student *t*-test contrasts showed that FMS patients reported significantly higher levels of pain (PNS: t(58) = 16.773, *p* < 0.001); fatigue (FNS: t(58) = 7.636, *p* < 0.001); depressive symptoms (BDI total score: t(58) = 5.670, *p* < 0.001); trait anxiety (STAI-T total score: t(58) = 5.017, *p* < 0.001); and sleeping disturbances (PSQI sleep disturbance score: t(58) = 4.894, *p* < 0.001) compared to healthy individuals. Patient scores for worrying about pain were also higher in FMS than in HC, but the differences did not reach statistical significance (PCS: t(58) = 1.687, *p* > 0.05) (full data are available in Table 1).

### 3.2. Group Differences in Neuropsychological Measures

**Figure 1:**
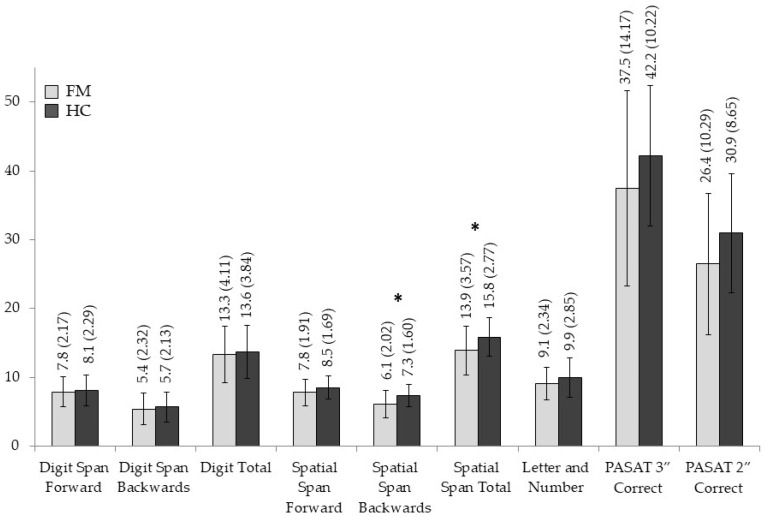
Group differences in neuropsychological measures of working memory. Means and standard deviations are shown for FMS patients and HC, with significant results marked with an asterisk.

**Figure 2:**
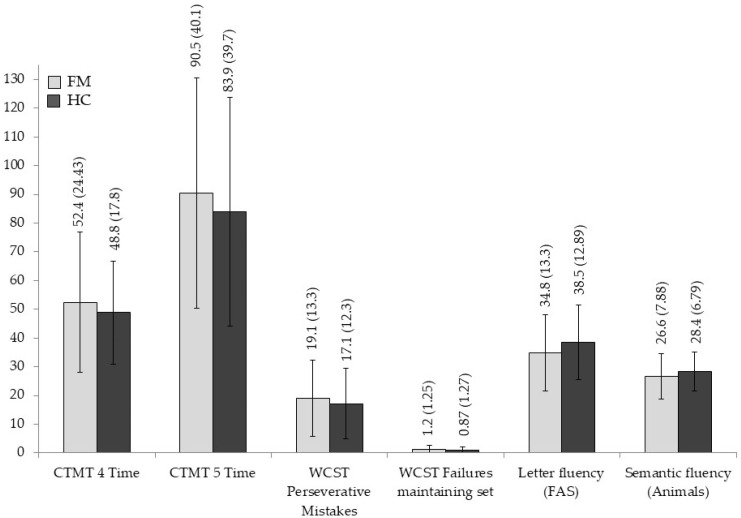
Group differences in neuropsychological measures of mental flexibility and access to memory information. Means and standard deviations are shown for fibromyalgia patients and healthy controls.

**Figure 3:**
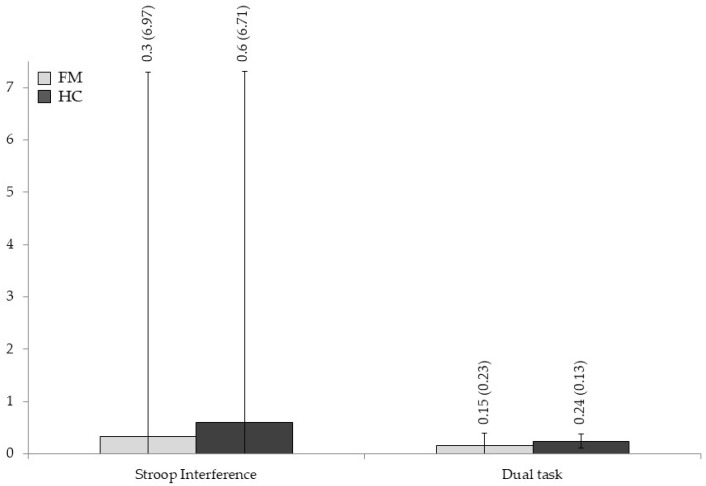
Group differences in neuropsychological measures of inhibition/interference control and the dual task. Means and standard deviations are shown for fibromyalgia patients and healthy controls.

**Figure 4:**
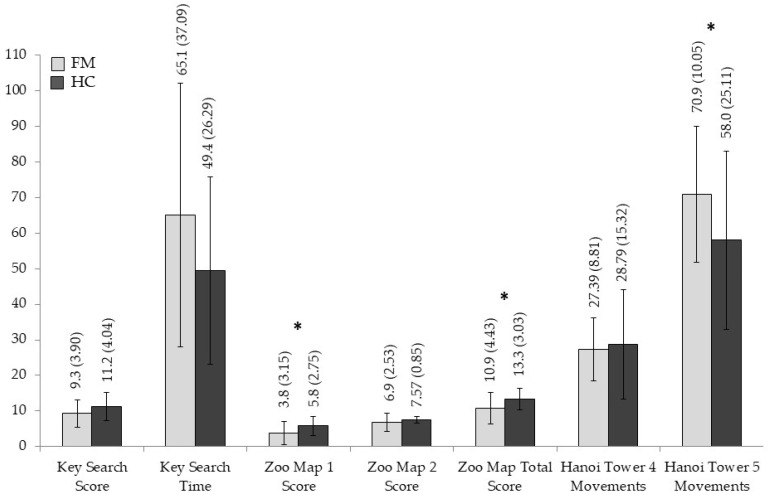
Group differences in neuropsychological measures for planning and problem-solving (high-order EFs). Means and standard deviations are shown for fibromyalgia patients and healthy controls, with significant results marked with an asterisk.

The means and standard deviations for each neuropsychological measure are displayed in Figure 1, Figure 2, Figure 3 and Figure 4, and significant statistical differences (the results of MANOVAs) are signaled with asterisks (the full data are available in Appendix A, Table A1).

Analyses on WM tests assessing the maintenance, manipulation, or updating of information reflected worse scores for the FMS group in most of the measures compared to HC individuals. However, differences only reached statistical significance for the SSBW score (F _[1,58]_ 7.208, *p* = 0.009, η^2^_p_ = 0.111) and the Spatial Span total score (F _[1,58]_ 5.663, *p* = 0.021, η^2^_p_ = 0.089), with size effects from moderate to high and moderate, respectively. The outcomes for tasks evaluating inhibition/interference control, mental flexibility/shifting, access to lexical memory, and the dual task also showed worse performances for the FMS patients, but none of them yielded significant effects (all *p* > 0.05).

MANOVAs conducted on complex EFs (planning and problem-solving tests) again demonstrated that FMS patients consistently performed lower than control individuals, except for ToH4D movements. Comparisons between groups evidenced significant differences in the performance of version 1 of the ZMT with a moderate-to-large size effect (F _[1,58]_ 6.829, *p* = 0.011, η^2^_p_ = 0.105), as well as the ZMT total score (F _[1,58]_ 6.319, *p* = 0.015, η^2^_p_ = 0.098) and the ToH5D movements (F _[1,58]_ 5.079, *p* = 0.028, η^2^_p_ = 0.081), both with moderate size effects. Nevertheless, we found no significant effects for comparisons based on KST score, KST time, and ZMT2 score (*p* > 0.05).

In order to explore the potential effects of clinical variables on the differences between groups on cognitive performance, ANCOVAs were performed, including those symptoms where statistical differences were observed between patients and healthy participants. Lower scores exhibited by FMS patients compared to HC individuals on every cognitive test were no longer different when pain, fatigue, and sleep disturbance scores were introduced individually as covariates. Only the ZMT total score and ToH5Dc movements survived the effect of fatigue and sleep disturbances. Regarding emotional symptoms, differences between groups in ZMT1, ToH5D movements, and SSBW persisted, even after controlling for the potential influences of anxiety and depression (full statistical details can be seen in Table 2).

### 3.3. Predictive Value of Simple EF on Planning and Problem-Solving

**Figure 5:**
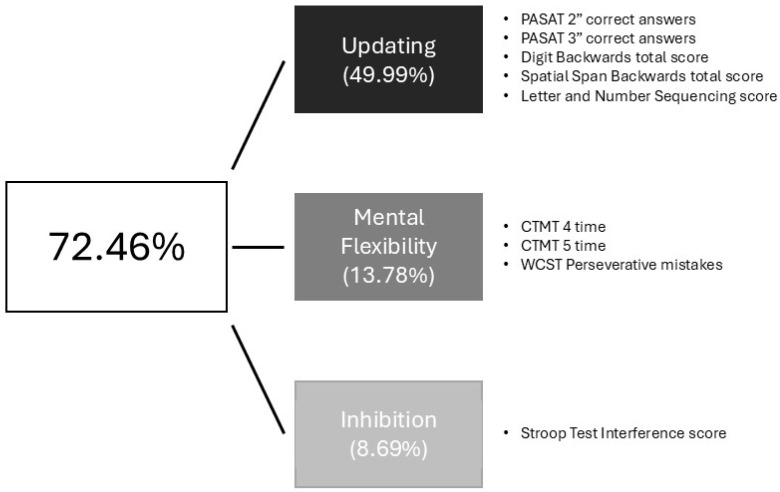
Schematic representation of the confirmatory factorial analysis. Percentages of explained variance by each factor are shown in parentheses. Measures that are saturated in each factor are also included.

The exploratory factorial analysis (EFA) using Principal Component Analysis (PCA) with Varimax rotation and Kaiser normalization for the extraction of main components provided a Kaiser–Meyer–Olkin (KMO) index of 0.820 and a significant Bartlett’s test of Sphericity χ^2^(45) = 250.70, *p* < 0.001, indicating the suitability of our data and the appropriateness of conducting a factor analysis. In the anti-image correlation data, we observed that the value associated with WCST failures to maintain set (0.306^a^) was low and far from the rest of the measures, so we decided to perform a confirmatory factor analysis, removing that variable from the three predetermined factors as obtained in the EFA. This new analysis yielded a KMO value of 0.839 and a Bartlett’s test of χ^2^(36) = 243.08, *p* < 0.001, with values bigger than 0.700 for all variables represented in the anti-image matrix, and three factors that explained 72.47% of the accumulated variance (see Figure 5). According to these results, we created three indexes by summing Z scores from the tests loading in each factor. These new composite indexes were introduced concurrently in multiple linear regression analyses employing the “Enter” method as independent variables for exploring their predictive role for complex EF performance, separately for each group of participants, as shown in Table 3.

**Figure 6:**
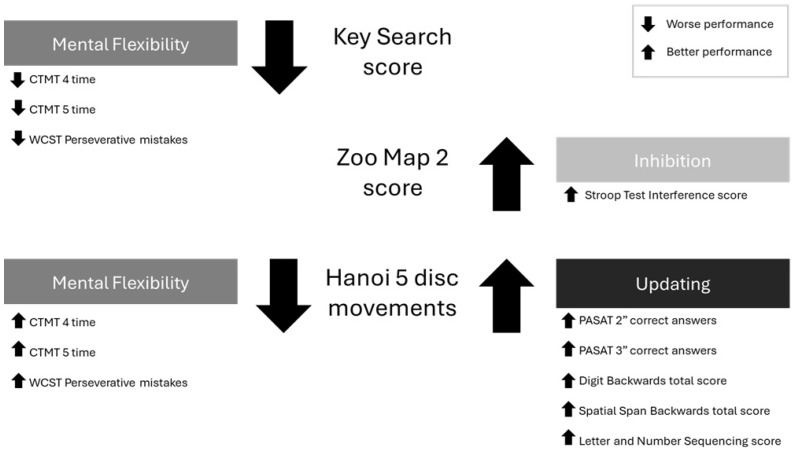
Results of the multiple regression analyses in the FMS group. Predictors of performance in each high-order EF are presented on both sides, together with arrows indicating their predicting direction.

Multiple regression analyses conducted for FMS patients (see Figure 6) showed that the mental flexibility index predicted the KST score (β = −0.599, *p* = 0.012), indicating that more time spent in CTMT 4 and CTMT 5, as well as more perseverative mistakes in the WCST, predicted a worse performance in the KST. The inhibition index was found to be a significant predictor for the ZMT2 score (β = 0.521, *p* = 0.003), where a better score in the SIC predicted a higher outcome in the ZMT2 test. Finally, both the mental flexibility and updating indices successfully predicted the performance in the ToH5D movements (β = −0.667, *p* = 0.010; β = −0.722, *p* = 0.006). More specifically, the better outcome in the updating index predicted better performance in the ToH5D test, where fewer movements were executed. Unexpectedly, we found that a worse performance in the ToH5D test (reflected in a higher number of movements to finish the task) was predicted by a better result in mental flexibility (less time and a lower number of mistakes made). Analyses performed by the HC group did not identify any index as a significant predictor for the complex EF tests. Full data for FMS and HC samples are available in Table 3.

## 4. Discussion

In the present investigation, we explored the presence of alterations in high-order cognitive functions, such as planning and problem-solving in FMS and, most critically, how impairments in those functions might be explained by the performance of the simple executive components (updating, inhibition, and mental flexibility) proposed by Miyake and colleagues [26,74]. Consistent with previous studies exploring executive functioning in FMS, we observed that patients scored lower than HC participants in a wide variety of neuropsychological tests, especially in those assessing visuospatial WM and the ability to plan and solve complex problems. Interestingly, and, as far as we are concerned, innovatively, multiple regression analyses revealed that tests involving updating, inhibition, and mental flexibility components partly predicted how patients behaved on high-order executive tests.

As indicated in the Section 3, we found neuropsychological discrepancies between FMS patients and HC participants in high-order EFs. Patients performed worse in tests measuring planning and problem-solving, though statistical relevance appeared only for the ZMT1 score, ZMT total score, and ToH5D. A detailed analysis of these tests demonstrates that patients showed impairments in the most difficult versions of both instruments, which could be interpreted as due to a higher processing demand [75,76]. Although this effect was still present after considering anxiety and depressive symptoms, differences between groups in the ZMT1 score and ToH5D disappeared when pain, fatigue, and sleep disturbances were included as covariates. These instruments have not been widely employed yet in neuropsychological research or in FMS, but the available data would be aligned with current data, at least partially, regarding the influence of pain and related symptoms on executive functioning measured by the ZMT [15,16,17]. In this vein, other authors reporting alterations in decision-making (other high-order cognitive function) in FMS also found a significant impact of pain [13,22] and fatigue [12] on cognitive performance without the influence of emotional variables such as anxiety or depression [12,14,22]. The fact that the present sample of patients was not taking opioid drugs or anticonvulsants commonly used to relieve pain in FMS could be a critical issue, as their pain levels could remain stable, thus indicating a greater impact on patients’ cognition, especially in tests with higher mental workload [75,77]. Regarding emotional symptoms, though our FMS sample scored significantly higher than controls in STAI-T and BDI scales, their levels of symptomatology are considered mild for depression [78,79] and medium for trait anxiety [71]. This is something that possibly differentiates our sample from others scoring higher on the same questionnaires [14,15] and also could be underpinning the scarce influence of these variables on our higher-order EF outcomes.

Measurements of simple executive components (updating, inhibition, and mental flexibility) exhibited lower scores for FMS in neuropsychological tests involving these cognitive abilities compared to HC participants. Specifically, the most concrete differences were observed in the SSBW, as a measure of updating processes in visuospatial WM, though pain, fatigue, sleep disturbances, and depression had a relevant effect on this performance, as previously reported [9,30]. However, performance in other updating tasks, as well as in inhibition and mental flexibility tests, was comparable between groups. Given that inhibition is considered to be one of the most clearly impaired functions in FMS [10], it was unexpected that we somehow found no significant differences in the Stroop test interference score, though there is some evidence pertaining to this effect [43]. Although FMS participants scored lower than HC individuals, the disparities were not significant in measures of mental flexibility, in accordance with studies employing Trail-Making Test-type instruments [33,35] or the WCST [12,80]. Furthermore, when taking into account studies that analyzed pain, fatigue, and emotional problems, their roles are not completely defined when considering data both for [5,34,81,82] and against their influences [15,24]. In sum, prior evidence seems to provide some support for the presence of impairments in simple EFs, but the variability in the reports is also remarkable. One reason for this might be that the tasks usually employed to assess them are not adequate, as they are sometimes mediated by mental speed, as in the TMT, or because they involve many other cognitive processes, as has been noted for the WCST [74].

On another level, our results complement and extend prior findings on the neuropsychological impairments of FMS patients in several aspects. As described above, the literature has focused on separately determining FMS performance in simple executive processes, as defined by Miyake’s model, and high-order EFs, meaning that the influences of the former on the latter have so far remained understudied. Due to the scarcity of research about this issue in FMS, the identification of the current findings with the existing literature may not be straightforward, and other clinical conditions and populations should be considered. Multiple regression analyses yielded statistically significant effects, suggesting to some extent that simple EFs successfully predicted the performance in high-order functions, but this was only true for FMS patients. This would support the idea of a hierarchical structure for the different EFs, reflecting models derived from different theoretical proposals [26,28], and is in accordance with recent investigations [83]. Specifically, we observed that greater performance in mental flexibility predicted better outcomes in planning, as shown by the KST. The influence of mental flexibility (measured with the Trail-Making Test and the WCST) on planning and problem-solving has recently been reported in participants with mild cognitive impairment [47]. Other investigations conducted in children have also highlighted a predictive role of mental flexibility on planning and problem-solving when using tower tests or science problems [49,84]. Thus, the literature would only partly be aligned with our findings in the KST as a planning activity, but would contrast with patient outcomes in the ToH5D, where we found an inverse relationship between mental flexibility (worse performance) associated with better performance (fewer movements). Understanding these findings requires an analysis of both high-order tasks’ particularities. Mental flexibility requirements would be important in both tasks in order to shift between possible sub-goals or solutions before choosing one, which has been described by Cooper and Marsh [85]; in contrast, ToH5D is longer than KST, presenting more intermediate sub-goals until its conclusion, and a greater amount of monitoring ability is required (associated with updating capacity following Miyake’s model). On one hand, the importance of monitoring behavior on ToH effectiveness was initially reported in children [86], and updating relevance in planning and problem-solving (high-order functions) has also been demonstrated by empirical studies, in which the authors found that better verbal and non-verbal WM turned out to be predictors of a smaller number of movements in tower-type tasks [83,87,88]. On the other hand, it has been reported that worse mental flexibility could be necessary for goal maintenance in executive control tasks [89]. As a result, it may be possible that FMS patients presenting with worse mental flexibility could show a better capacity to memorize the selected strategy, together with a better ability to monitor their ongoing behavior (as a WM process), thus needing fewer movements to finish the ToH5D task.

Remarkably, as a final result, multiple regression analyses showed that greater inhibition capabilities predicted superior outcomes in the ZMT2 score in FMS. This test asks the participants to follow a set of given instructions and is a much easier way to assess their capacity for problem-solving, though it requires the ability to select the correct places to visit instead of others that could act as distractions. As this test is applied immediately after the first part (ZMT1), it sometimes also requires the subject to disregard the first solution they devised in favor of the second one already provided by the instructions [18]. Using this instrument, Oosterman and coworkers [90] suggested the relevance of inhibition in order to obtain a successful result in the ZMT. Besides this, the fact that no predictors were found for the ZMT1 was striking. Prior research has reported controversial conclusions regarding this test, as sometimes, it has been defined as an ecological instrument for the assessment of planning and problem-solving [91], but other investigations have described it as not as specific as the ZMT2 score for discriminating frontal damage [92]. In addition, as a more complex form of the task, it may be necessary to incorporate other aspects of cognition that could act as better predictors, such as episodic memory, spatial perception, or mental speed, which were not considered in the present study.

Our research demonstrates some strengths, such as pharmacological restrictions and other broad exclusion criteria, together with a comprehensive neuropsychological assessment of EF with varying complexity, which could be related to some of the inconsistencies found when compared with other studies. Nevertheless, it also presents some limitations that should be considered. We need to highlight the source of the FMS sample, as the participants were recruited from a local patients’ association where they are offered physical and psychological interventions (psychoeducation about FMS and cognitive-behavioral techniques for pain and other symptoms management). This could explain the absence of differences with HC in terms of worrying about pain and the low levels of depressive symptoms in our clinical participants compared to the general population, as pain rumination, magnification, and helplessness are frequently targets of psychotherapy in FMS. Another limitation to consider refers to the instruments employed, as most of them are designed to evaluate patients with more severe brain damage. This could make them less sensitive to dyscognition in chronic pain, considering that these patients are usually unsuccessful in the high-cognitive-load tasks. In any case, the same or similar neuropsychological and cognitive tests have been widely employed by other researchers who reported results both in accordance with and differing from ours in FMS patients and in other clinical populations.

## 5. Conclusions

The results reported in this study are the first to provide some support to the idea that high-order EFs in FMS patients can be partially determined by the under-functioning of simple executive processes. These findings have clinical relevance, though they still need to be replicated and supported by future research, as they could help in guiding a better comprehension of FMS patients’ needs and also may serve as initial guidelines for the design and the development of neuropsychological rehabilitation programs oriented to improve EF abilities in a comprehensive way, including not only the more demanding higher-level processes but also easier ones that seem to have some influence on the abilities. Consequently, it would lead to finding a more adaptive way for FMS patients to cope with symptoms and everyday activities.

We also would like to delineate some other future lines of research to further explore planning and problem-solving abilities in FMS, as examples of high-order EFs. The potential and mutual influences between EF and intelligence have already been studied (see Dugan and García-Barrera [93] for a review). Some authors have described that fluid intelligence is related to some extent to different EF processes [94], but until now, no investigation has addressed this in FMS patients. Further research is required to analyze the different factors that may alter high-level executive processes in people suffering from fibromyalgia.

## Figures and Tables

**Table 1:** Demographic and clinical variables of FMS and HC samples. Means and standard deviations (in parenthesis) of age, estimated IQ, tobacco and alcohol consumption, pain and fatigue levels, depressive symptoms, trait anxiety, pain worrying, and sleep disturbances. Number of participants for marital status, educational level, medication, tobacco, and alcohol intake appear also in percentages (in parenthesis). Significant differences are shown in bold.

	FMS Patients N = 30	HC Participants N = 30	t/x^2^	*p*-Value
Age (M ± SD)	49.73 (9.09)	48.60 (8.50)	0.487	0.628
Marital status (N/%)			3.498	0.321
Married	21 (70)	17 (56.66)		
Single	1 (3.33)	5 (16.67)		
Widow	1 (3.33)	2 (6.67)		
Separated/Divorced	7 (23.34)	6 (20)		
Educational level (N/%)			1.529	0.821
Elemental education	1 (3.33)	1 (3.33)		
Primary Education	6 (20)	3 (10)		
Basic Education	10 (33.33)	10 (33.33)		
High School	7 (23.34)	10 (33.33)		
Higher Education	6 (20)	6 (20)		
Estimated Intelligence Quotient (M ± SD)				
Vocabulary	40.97 (10.13)	44.37 (8.05)	−1.439	0.155
Block Design	35.40 (12.82)	38.03 (9.17)	−0.915	0.364
Tobacco consumers (N/%)			0.341	0.559
Yes	7 (23.33)	9 (30)		
No	23 (76.67)	21 (70)		
Cigarettes/day (M ± SD)	3.43 (6.76)	4.27 (8.67)	−0.415	0.680
Alcohol consumers (N/%)			7.177	**0.007**
Yes	6 (20)	16 (53.33)		
No	24 (80)	14 (46.67)		
Alcoholic drinks/day (M ± SD)	0.37 (0.89)	0.89 (1.21)	−1.902	0.062
Years since diagnosis (M ± SD)	6.97 (5.16)			
Medication (N/%)				
NSAIDs	16 (53.33)	0	21.818	**0.000**
SSRIs	5 (16.67)	1 (3.33)	2.963	0.085
Dual ADs	4 (13.33)	0	4.286	**0.038**
Non-Opioid Analgesics	12 (40)	0	15.000	**0.000**
Dopamine Agonists	1 (3.33)	0	1.017	0.313
Other	9 (30)	1 (3.33)	7.680	**0.006**
Pain level (PNS) (M ± SD)	7.71 (1.42)	1.63 (1.13)	16.773	**0.000**
Fatigue level (FNS) (M ± SD)	5.60 (2.42)	1.73 (1.36)	7.636	**0.000**
Depressive symptoms (BDI) (M ± SD)	16.70 (9.11)	5.70 (5.48)	5.670	**0.000**
Trait anxiety (STAI-T) (M ± SD)	29.90 (11.27)	16.47 (9.39)	5.017	**0.000**
Pain worrying (PCS) (M ± SD)	21.10 (1.33)	15.83 (11.58)	1.687	0.097
Sleep disturbances (PSQI) (M ± SD)	1.70 (0.57)	0.91 (0.62)	5.200	**0.000**

NSAIDs: Non-steroidal anti-inflammatory drugs; SSRIs: Selective serotonin reuptake inhibitors; Dual ADs: Dual anti-depressants; Other includes antihypertensives, anticholesterolemic agents, antihistamines, occasional quetiapine, stomach protectors, anti-arthrosics, antivertiginous, and glucocorticoids; PNS: Pain Numerical Scale; FNS: Fatigue Numerical Scale; BDI: Beck Depression Inventory; STAI-T: State-Trait Anxiety Inventory-Trait Form; PCS: Pain Catastrophizing Scale; PSQI: Pittsburgh Sleep Quality Index.

**Table 2:** ANCOVAs of simple and high-order EF measures, including clinical symptoms as covariates. Statistically significant results are marked in bold, and their size effects are also reported.

	Pain Effect (d.f. = 1, 57)	Fatigue Effect (d.f. = 1, 57)	Sleeping Disturbances Effect (d.f. = 1, 57)	Depression Effect (d.f. = 1, 57)	Anxiety Effect (d.f. = 1, 57)
***WM (maintaining, manipulating, updating)***					
Spatial Span Backwards Score	F = 0.86; *p* = 0.358	F = 1.205; *p* = 0.277	F = 0.513; *p* = 0.477	F = 1.892; *p* = 0.174	**F = 5.182;** ***p* = 0.027,** **η^2^_p_ = 0.083**
Spatial Span Total Score	F = 0.744; *p* = 0.392	F = 0.824; *p* = 0.368	F = 0.594; *p* = 0.444	F = 1.021; *p* = 0.316	F = 3.039; *p* = 0.087
***Planning and problem-solving***					
Zoo Map 1 Score	F = 0.619; *p* = 0.435	F = 3.405; *p* = 0.070	F = 3.662; *p* = 0.061	**F = 4.897;** ***p* = 0.031,** **η^2^_p_ = 0.079**	**F = 4.794;** ***p* = 0.033,** **η^2^_p_ = 0.078**
Zoo Map Total Score	F = 0.261; *p* = 0.611	**F = 4.657;** ***p* = 0.035,** **η^2^_p_ = 0.076**	F = 3.541; *p* = 0.065	F = 2.923; *p* = 0.093	F = 3.578; *p* = 0.064
Hanoi Tower 5 Disc Movements	F = 0.477; *p* = 0.493	F = 1.943; *p* = 0.169	**F = 5.513;** ***p* = 0.022,** **η^2^_p_ = 0.088**	**F = 4.882;** ***p* = 0.031,** **η^2^_p_ = 0.079**	**F = 4.608;** ***p* = 0.036,** **η^2^_p_ = 0.075**

**Table 3:** Multiple regression analyses including simple EF processes as possible predictors of performance in high-order EF for FMS and HC samples.

Complex EF Test		Fibromyalgia Patients						Healthy Controls					
Key Search Score	*Model*	*R*	*r* ^2^	*SE*	*F*	*d.f.*	*p*	*R*	*r* ^2^	*SE*	*F*	*d.f.*	*p*
	1	0.622	0.387	0.794	5.466	(3,26)	0.005	0.420	0.177	0.953	1.859	(3,26)	0.161
	*Predictors*	*Β*	*SE*	*95% CI*	*β*	*t*	*p*	*Β*	*SE*	*95% CI*	*β*	*t*	*p*
	Constant	−0.198	0.150	[−0.507, 0.111]		−1.317	0.199	0.187	0.183	[−0.188, 0.563]		1.026	0.314
	Mental Flexibility	−0.218	0.080	[−0.383, −0.053]	−0.599	−2.710	0.012	−0.168	0.108	[−0.391, 0.055]	−0.387	−1.548	0.134
	Inhibition	0.218	0.146	[−0.082, 0.518]	0.233	1.492	0.148	0.022	0.194	[−0.378, 0.421]	0.022	0.112	0.912
	Updating	−0.017	0.052	[−0.123, 0.089]	−0.071	−0.324	0.748	0.011	0.073	[−0.139, 0.162]	0.040	0.151	0.881
Zoo Map 1 Score	*Model*	*R*	*r* ^2^	*SE*	*F*	*d.f.*	*p*	*R*	*r* ^2^	*SE*	*F*	*d.f.*	*p*
	1	0.266	0.071	1.033	0.661	(3,26)	0.584	0.556	0.309	0.779	3.869	(3,26)	0.021
	*Predictors*	*Β*	*SE*	*95% CI*	*β*	*t*	*p*	*Β*	*SE*	*95% CI*	*β*	*t*	*p*
	Constant	−0.316	0.196	[−0.718, 0.086]		−1.617	0.118	0.257	0.149	[−0.050, 0.564]		1.718	0.098
	Mental Flexibility	−0.128	0.105	[−0.343, 0.086]	−0.334	−1.229	0.230	−0.169	0.089	[−0.351, 0.014]	−0.436	−1.902	0.068
	Inhibition	−0.046	0.190	[−0.436, 0.345]	−0.046	−0.240	0.812	−0.298	0.159	[−0.625, 0.028]	−0.332	−1.877	0.072
	Updating	−0.027	0.067	[−0.165, 0.111]	−0.109	−0.401	0.692	0.028	0.060	[−0.095, 0.151]	0.113	0.466	0.645
Zoo Map 2 Score	*Model*	*R*	*r* ^2^	*SE*	*F*	*d.f.*	*p*	*R*	*r* ^2^	*SE*	*F*	*d.f.*	*p*
	1	0.606	0.368	1.116	5.039	(3,26)	0.007	0.276	0.076	0.456	0.717	(3,26)	0.551
	*Predictors*	*Β*	*SE*	*95% CI*	*β*	*t*	*p*	*Β*	*SE*	*95% CI*	*β*	*t*	*p*
	Constant	−0.084	0.211	[−0.519, 0.350]		−0.399	0.693	0.194	0.087	[0.014, 0.374]		2.215	0.036
	Mental Flexibility	−0.068	0.113	[−0.300, 0.164]	−0.135	−0.599	0.554	−0.074	0.052	[−0.181, 0.033]	−0.377	−1.425	0.166
	Inhibition	0.674	0.205	[0.253, 1.096]	0.521	3.288	0.003	−0.002	0.093	[−0.193, 0.189]	−0.004	−0.022	0.983
	Updating	0.060	0.073	[−0.089, 0.209]	0.185	0.828	0.415	−0.039	0.035	[−0.111, 0.034]	−0.309	−1.099	0.282
Hanoi Tower 4 Discs Movements	*Model*	*R*	*r* ^2^	*SE*	*F*	*d.f.*	*p*	*R*	*r* ^2^	*SE*	*F*	*d.f.*	*p*
	1	0.322	0.104	0.709	1.001	(3,26)	0.408	0.226	0.051	1.269	0.468	(3,26)	0.707
	*Predictors*	*Β*	*SE*	*95% CI*	*β*	*t*	*p*	*Β*	*SE*	*95% CI*	*β*	*t*	*p*
	Constant	−0.055	0.134	[−0.331, 0.221]		−0.408	0.687	0.011	0.243	[−0.489, 0.511]		0.044	0.965
	Mental Flexibility	0.053	0.072	[−0.094, 0.201]	0.199	0.745	0.463	0.057	0.144	[−0.239, 0.354]	0.107	0.398	0.694
	Inhibition	−0.184	0.130	[−0.452, 0.084]	−0.266	−1.409	0.171	−0.297	0.259	[−0.829, 0.235]	−0.238	−1.146	0.262
	Updating	0.020	0.046	[−0.075, 0.114]	0.113	0.423	0.676	0.067	0.098	[−0.133, 0.268]	0.197	0.691	0.496
Hanoi Tower 5 Discs Movements	*Model*	*R*	*r^2^*	*SE*	*F*	*d.f.*	*p*	*R*	*r^2^*	*SE*	*F*	*d.f.*	*p*
	1	0.525	0.275	0.743	3.292	(3,26)	0.036	0.289	0.083	1.101	0.789	(3,26)	0.511
	*Predictors*	*Β*	*SE*	*95% CI*	*β*	*t*	*p*	*Β*	*SE*	*95% CI*	*β*	*t*	*p*
	Constant	0.190	0.141	[−0.099, 0.479]		1.348	0.189	−0.312	0.211	[−0.746, 0.122]		−1.479	0.151
	Mental Flexibility	−0.208	0.075	[−0.363, −0.054]	−0.667	−2.775	0.010	−0.136	0.125	[−0.394, 0.121]	−0.287	−1.089	0.286
	Inhibition	−0.068	0.136	[−0.348, 0.213]	−0.084	−0.496	0.624	0.066	0.225	[−0.395, 0.528]	0.060	0.295	0.770
	Updating	−0.146	0.048	[−0.245, −0.046]	−0.722	−3.017	0.006	−0.006	0.085	[−0.180, 0.168]	−0.019	−0.068	0.946

*R*: coefficient R; *r*^2^: adjusted r-squared; *SE*: standard error; *d.f.*: degrees of freedom; *Β*: unstandardized beta; *CI*: confidence interval; *β*: standardized beta.

## Data Availability

The original data presented in the study are openly available in OSF at DOI 10.17605/OSF.IO/ANKSD, https://osf.io/anksd/ (accessed on 16 May 2025).

## Abbreviations

The following abbreviations are used in this manuscript: FMS: Fibromyalgia Syndrome; WM: Working Memory; EF: Executive Functions; BADS: Behavioural Assessment of the Dysexecutive Syndrome; R-SAT: Revised Strategy Application Test; ZMT: Zoo Map Test; KST: Key Search Test; IGT: Iowa Gambling Test; ToL: Tower of London; PASAT: Paced Auditory Serial Addition Test; HC: Healthy Controls; DSFW: Digit Span Forward; DSBW: Digit Span Backwards; WAIS-III: Wechsler Adult Intelligence Scale III; SSFW: Spatial Span Forward; SSBW: Spatial Span Backwards; WMS-III: Wechsler Memory Scale III; LNS: Letter and Number Sequencing; SIC: Stroop Interference Control; CTMT: Comprehensive Trail Making Test; WCST: Wisconsin Card Sorting Test; FAS: Letter Fluency Task; DT: Dual Task; ToH: Tower of Hanoi; ToH4D: Tower of Hanoi 4 Discs; ToH5D: Tower of Hanoi 5 Discs; PNS: Pain Numerical Scale; FNS: Fatigue Numerical Scale; BDI: Beck Depression Inventory; STAI-S: State-Trait Anxiety Scale State; STAI-T: State-Trait Anxiety Scale Trait; PSQI: Pittsburg Sleep Quality Index; PCS: Pain Catastrophizing Scale; *d.f.*: degrees of freedom; EFA: Exploratory Factorial Analysis; PCA: Principal Component Analysis; KMO: Kaiser-Meyer-Olkin.

## Appendix A

**Table A1:** One factor ANOVA of neuropsychological measures of WM, simple and complex EF. Means and standard deviations for fibromyalgia patients are reported along with the statistic F, statistical significance values, and size effects. Significant results appear in bold.

	FMS N = 30 (Mean/SD)	HC N = 30 (Mean/SD)	F for Group	*p* for Group	η^2^_p_ for Group
*WM (maintaining, manipulating, updating*)					
Digit Span Forward Score	7.86 (2.177)	8.10 (2.294)	0.163	0.688	0.003
Digit Span Backwards Score	5.40 (2.328)	5.70 (2.135)	0.270	0.605	0.005
Digit Total Score	13.30 (4.112)	13.67 (3.845)	0.127	0.723	0.002
Spatial Span Forward Score	7.80 (1.919)	8.50 (1.697)	2.240	0.140	0.037
Spatial Span Backwards Score	6.10 (2.023)	7.37 (1.608)	7.208	**0.009**	0.111
Spatial Span Total Score	13.90 (3.575)	15.87 (2.776)	5.663	**0.021**	0.089
Letter and Number	9.07 (2.348)	9.97 (2.859)	1.776	0.188	0.030
PASAT 3” Correct	37.50 (14.176)	42.20 (10.226)	2.169	0.146	0.036
PASAT 2” Correct	26.47 (10.298)	30.97 (8.656)	3.357	0.072	0.55
*Inhibition/interference control*					
Stroop Interference	0.33 (6.970)	0.61 (6.714)	0.024	0.878	0.000
*Access to memory information*					
Letter fluency (FAS)	34.80 (13.309)	38.53 (12.899)	1.217	0.274	0.021
Semantic fluency (Animals)	26.63 (7.885)	28.47 (6.791)	0.931	0.339	0.016
*Mental flexibility/Shifting*					
CTMT 4 Time	52.43 (24.384)	48.83 (17.825)	0.426	0.516	0.007
CTMT 5 Time	90.56 (40.098)	83.93 (39.773)	0.413	0.523	0.007
WCST Perseverative Mistakes	19.07 (13.362)	17.10 (12.319)	0.480	0.491	0.008
WCST Failures maintaining set	1.27 (1.258)	0.87 (1.279)	1.491	0.227	0.025
*Dual task*	0.15 (0.236)	0.24 (0.137)	3.359	0.072	0.055
*Planning and problem solving*					
Key Search Score	9.30 (3.905)	11.27 (4.042)	3.673	0.060	0.060
Key Search Time	65.03 (37.096)	49.47 (26.296)	3.294	0.075	0.054
Zoo Map 1 Score	3.80 (3.156)	5.80 (2.759)	6.829	**0.011**	0.105
Zoo Map 2 Score	6.90 (2.537)	7.57 (0.858)	1.858	0.178	0.031
Zoo Map Total Score	10.90 (4.436)	13.37 (3.034)	6.319	**0.015**	0.098
Hanoi Tower 4 Disc Movements	27.39 (8.814)	28.79 (15.325)	0.190	0.665	0.003
Hanoi Tower 5 Disc Movements	70.97 (19.059)	58.00 (25.118)	5.079	**0.028**	0.081
